# Ecology and functions of *Trichoderma* in coffee and cocoa agroecosystems: bibliometric and systematic insights for sustainable agriculture

**DOI:** 10.3389/fmicb.2025.1717484

**Published:** 2025-11-28

**Authors:** Henry W. Santillan-Culquimboz, Santos Triunfo Leiva, Milagros Ninoska Munoz-Salas, Wagner Meza-Maicelo, Flavio Lozano-Isla, Manuel Oliva-Cruz, César R. Balcázar-Zumaeta

**Affiliations:** 1 Instituto de Investigación para el Desarrollo Sustentable de Ceja de Selva (INDES-CES), Universidad Nacional Toribio Rodríguez de Mendoza de Amazonas, Chachapoyas, Peru; 2 Centro de Investigación e Innovación en Granos y Semillas, Universidad Nacional Toribio Rodríguez de Mendoza de Amazonas (UNTRM), Chachapoyas, Amazonas, Peru; 3 Facultad de Ingeniería y Ciencias Agrarias, Universidad Nacional Toribio Rodríguez de Mendoza de Amazonas (UNTRM), Chachapoyas, Amazonas, Peru; 4 Department of Earth and Environment, Institute of Environment, Florida International University, Miami, FL, United States; 5 Instituto de Investigación, Innovación y Desarrollo para el Sector Agrario y Agroindustrial (IIDAA), Facultad de Ingeniería y Ciencias Agrarias, Universidad Nacional Toribio Rodríguez de Mendoza de Amazonas, Chachapoyas, Peru

**Keywords:** biological control, bioremediation, endophytes, entomopathogenic fungi, fungal diversity, nutrient solubilization, systemic resistance

## Abstract

Coffee and cacao are strategic tropical crops increasingly threatened by climate change, soil degradation, and disease outbreaks. In this context, *Trichoderma* has emerged as a multifunctional microorganism with significant ecological roles and biotechnological applications. This study aimed to comprehensively assess the functions of *Trichoderma* in coffee (*Coffea* spp.) and cacao (*Theobroma cacao*) agroecosystems using a combined bibliometric and systematic review approach. Bibliometric analyses of 266 documents indexed in Scopus and Web of Science (1985–2025) revealed sustained publication growth, with Latin America and the Asia-Pacific region as leading contributors. Six major thematic clusters were identified, encompassing biological control, plant growth promotion, biotechnology, and fungal diversity. The systematic review confirmed that *Trichoderma* colonizes diverse ecological niches (rhizosphere, endosphere, phyllosphere) and acts through mycoparasitism, antibiosis, nutrient solubilization, and induced systemic resistance. Evidence highlights its effectiveness against key pathogens (*Moniliophthora* spp., *Phytophthora* spp., *Hemileia vastatrix*) and its contribution to host growth and stress tolerance. Less explored applications include heavy-metal bioremediation, waste bioconversion, mycorrhizal interactions, and entomopathogenic potential. However, gaps remain regarding strain variability under field conditions and underexplored avenues such as cadmium remediation in cacao. Overall, the findings position *Trichoderma* as a cornerstone microbial resource for enhancing the resilience and sustainability of coffee and cacao agroecosystems.

## 1 Introduction

The socioeconomic impact of coffee (*Coffea* spp.) and cacao (*Theobroma cacao*) has been pivotal in the development of producing regions worldwide (Ballesteros Possú et al., 2021; Siles et al., 2022; Solarte-Guerrero et al., 2023). The coffee sector is estimated to generate over US$200 billion annually, with a growth rate of 2.2% (Oliva-Cruz et al., 2024). In turn, the global cocoa market is projected to reach US$1.33 trillion by 2027 (Kongor et al., 2024), establishing itself, alongside coffee, as one of the agricultural sectors with the highest economic dynamism and employment-generating potential worldwide.

The production of these crops is mainly concentrated in developing countries and has become a fundamental pillar of their local economies. In the case of coffee, Brazil leads global production, accounting for approximately 33% of the total, followed by Vietnam (12%), Colombia (10%), and Indonesia (7%) (Silva et al., 2023). With respect to cacao, West Africa accounts for approximately 40% of global production, led by Côte d’Ivoire and Ghana, and complemented by producers such as Indonesia, Brazil, and Ecuador (Kongor et al., 2024). It is worth highlighting that most of this production is in the hands of smallholder farmers, whose livelihoods depend directly on these crops, representing the means of subsistence for more than 30 million rural families (Jezeer et al., 2017).

Nevertheless, despite their global significance, factors such as climate change, soil degradation, and high susceptibility to pests and diseases compromise the stability of production (Kongor et al., 2024). In this context, production systems that respect the dynamics of natural ecosystems have gained increasing relevance as a key strategy to foster resilience and mitigate these risks (de Sousa et al., 2021). A clear example is the coffee and cacao production models, which are largely oriented toward organic production and associated with diverse tree species (Solarte-Guerrero et al., 2023; Oliva-Cruz et al., 2024). These systems promote key ecological services, such as soil conservation, the maintenance of fertility, and greater resistance to pests and diseases, which largely depend on microbial presence and associations (Sahoo et al., 2025; Vaupel et al., 2025).

Since the transition toward sustainable agriculture is closely linked to the management of this beneficial microbiota, agricultural practices exert a decisive influence on the composition and abundance of these microbial consortia, modulating their capacity to provide ecological services (Vaupel et al., 2025). The microorganism–soil–plant interaction is a complex and dynamic natural process on which essential biogeochemical processes for the balance of agroecosystems depend (Foley et al., 2024; Fontaine et al., 2024; Pascutti et al., 2024). Within this diversity, the genus *Trichoderma* has been extensively studied due to its multifaceted potential in promoting plant growth and providing protection against biotic and abiotic stress (Alizadeh et al., 2024).

Understanding the ecology and behavior of *Trichoderma* in coffee and cacao agroecosystems is essential to optimize its application in sustainable agriculture (Asghar et al., 2024). These cosmopolitan species stand out for their resilience to unfavorable environments, efficient nutrient utilization, and ability to modify the rhizosphere, in addition to exhibiting marked aggressiveness against plant pathogens (Contreras-Cornejo et al., 2016; Woo et al., 2023). This adaptability is supported by their high potential to synthesize and release enzymes such as cellulases, xylanases, and chitinases, as well as secondary metabolites, enabling them to decompose complex compounds and colonize diverse ecological niches, ranging from plant surfaces to intra- and intercellular spaces within plant tissues (Sánchez Hernández et al., 2018; Choez-Guaranda et al., 2023).

*Trichoderma*–plant interactions also activate defense signaling pathways. Colonization often triggers systemic acquired resistance (SAR), mediated by salicylic acid, and induced systemic resistance (ISR), associated with jasmonic acid and ethylene. Together, these hormonal responses fortify plant defense against necrotrophic and biotrophic pathogens (Saravanakumar et al., 2016; Singh et al., 2019; Yadav et al., 2019; Al-Zahrani et al., 2020). Direct antagonism against phytopathogens occurs through mycoparasitism, antibiosis, and competition for nutrients and space. These mechanisms have demonstrated effectiveness against major coffee and cacao pathogens, including *Moniliophthora roreri, M. perniciosa, Phytophthora palmivora,* and *Hemileia vastatrix* (de Sousa et al., 2021; Leiva et al., 2022; Reyes et al., 2023). Beyond disease suppression, *Trichoderma* spp. also promotes plant growth, improves nutrient uptake, and enhances tolerance to abiotic stresses such as drought, salinity, and heavy metal toxicity (Guzmán-Guzmán et al., 2025).

Beyond its roles in plant health, *Trichoderma* demonstrates versatility in applications relevant to sustainable agriculture. These include insect pest biocontrol, heavy-metal bioremediation, organic waste bioconversion, and synergistic interactions with mycorrhizal fungi (Amin et al., 2014; Tuesta-Pinedo et al., 2017; Gil et al., 2023). Such multifunctionality positions *Trichoderma* as a cornerstone microbial resource for reducing reliance on chemical inputs and strengthening the ecological foundations of agroecosystems. However, important challenges remain. The variability of *Trichoderma* strains under field conditions complicates their consistent application, and certain promising avenues, such as cadmium remediation in cacao systems, remain underexplored.

Addressing these knowledge gaps requires integrative research frameworks that encompass both the breadth of global scientific production and the depth of ecological mechanisms at the plant–microbe–pathogen interface. In this context, the present study adopts an integrative methodological approach that combines macro-level trend analysis with micro-level evidence synthesis. Bibliometric analysis enables a quantitative mapping of the temporal evolution, geographical distribution, and thematic structure of research (Donthu et al., 2021), while the systematic review qualitatively synthesizes ecological and functional mechanisms, ranging from plant growth promotion to biocontrol. The integration of these approaches provides a comprehensive and complementary perspective, allowing for the identification of critical knowledge gaps.

Unlike previous reviews that have examined the genus *Trichoderma* mainly from agronomic or biocontrol perspectives, the present study offers a novel and integrative framework focused exclusively on coffee and cacao agroecosystems. This dual approach bridges global research trends with the specific ecological mechanisms that underpin the genus’s multifunctionality. Furthermore, it highlights emerging and underexplored ecological roles including heavy metal bioremediation, agricultural residue bioconversion, and entomopathogenic potential thereby establishing a strong conceptual foundation for the development of biotechnological and management strategies aimed at enhancing the sustainability of tropical production systems.

Accordingly, this study aims to provide an integrative analysis of the ecological and functional roles of the genus *Trichoderma* in coffee (*Coffea* spp.) and cacao (*Theobroma cacao*) agroecosystems. Specifically, the objectives are to: (i) characterize the temporal evolution, thematic patterns, and geographical distribution of *Trichoderma* research in coffee and cacao agroecosystems using bibliometric indicators; (ii) synthesize scientific evidence on the mechanisms of action of *Trichoderma* in plant–microbe and microbe–pathogen interactions; and (iii) identify knowledge gaps and research opportunities to advance the sustainable management of coffee and cacao production systems.

## 2 Materials and methods

This study employed a mixed exploratory–descriptive methodological approach structured in two complementary stages. The first stage consisted of a bibliometric analysis aimed at quantitatively characterizing research on *Trichoderm*a in coffee and cacao production systems, thereby revealing thematic patterns that informed the subsequent stage. The second stage comprised a complementary systematic review to provide a comprehensive understanding of the ecology and functions of the genus *Trichoderma* in these agroecosystems.

### 2.1 Stage 1: extraction and processing of bibliometric data

#### 2.1.1 Data sources and search strategy

The Scopus and Web of Science databases were used to retrieve scientific literature related to the applications of the genus *Trichoderma* in coffee and cacao crops (Baas et al., 2020). These databases are preferred sources for bibliometric studies because they provide broad coverage and comprehensive metadata across multidisciplinary fields of research (Donthu et al., 2021; Mishra et al., 2024). To ensure complete and accurate retrieval, the following Boolean search equation was constructed: (Trichoderma) AND (Coffee OR Coffea OR Cacao OR Cocoa OR “*Theobroma cacao*”).

The search yielded an initial total of 264 documents in Scopus and 291 in Web of Science. The search covered documents published between 1985 and 20 June 2025, with no language restrictions, and included the categories “articles,” “reviews,” and “conference papers.” Additional filters were applied to exclude non-relevant subject areas such as neuroscience and veterinary sciences. All available bibliometric records from both databases were compiled and exported in RIS (Research Information Systems) format.

The data cleaning process involved removing duplicates using Zotero and conducting a comprehensive manual screening to exclude studies that were not aligned with the objectives of this research. As a result, a final dataset of 266 unique documents was obtained, comprising 98 studies focused on coffee and 168 on cacao.

#### 2.1.2 Method of analysis

The extraction, processing, and analysis of bibliometric data were conducted using Python (version 3.12.11), which enabled data cleaning, normalization, and statistical analysis of publications. The curated dataset was subsequently exported in RIS format for processing in VOSviewer (version 1.6.20), a specialized tool for constructing and visualizing bibliometric network maps.

Key indicators analyzed included scientific productivity, assessed by the number of publications per period and author; citation metrics and journal impact indicators, used to evaluate scientific influence; and thematic structure, examined through keyword co-occurrence analysis to identify research areas.

Visualizations were developed through an integrated approach combining statistical plots generated in Python (using the pandas, matplotlib, and seaborn libraries), bibliometric network maps constructed in VOSviewer, and complementary graphs generated in R with the ggplot2 package. The complete process of retrieval, curation, and analysis is summarized in Supplementary Figure S1.

### 2.2 Stage 2: systematic literature review

#### 2.2.1 Purpose and approach

The systematic review was designed as a qualitative complement to the bibliometric analysis, with the objective of deepening the understanding of the mechanisms of action and ecological functions of the genus *Trichoderma* in coffee and cacao cultivation systems. Methodological principles of qualitative thematic systematic reviews were applied and adapted to the context of biological and agroecological sciences (Bearman et al., 2012).

This stage addressed the research question: What are the mechanisms of action and ecological functions of *Trichoderma* reported in the scientific literature, and how are they related to documented applications in coffee and cacao crops?

The approach justified the use of a search strategy without crop restriction, given that the mechanisms of action of *Trichoderma* are generally conserved across different agricultural systems. Such a comprehensive perspective enables better interpretation of the specific applications in coffee and cacao identified through the bibliometric analysis.

#### 2.2.2 Literature retrieval

The literature retrieval process was structured through specific criteria for searching, selecting, and evaluating studies in Scopus and Web of Science, without restriction by crop (query performed on 20 June 2025). The thematic axes identified in the bibliometric analysis - focused on the ecology and functional mechanisms of *Trichoderma* in agroecosystems - were used to develop targeted search strategies, particularly emphasizing microbe–plant–pathogen interactions.

Based on these criteria, multiple search equations were established, each covering a distinct dimension of biological activity. The complete set of thematic search strategies is presented in Table 1.

**Table 1:** Thematic search strategies applied to the systematic review of *Trichoderma* spp.

Thematic axis	Search equation
Species diversity	(trichoderma* AND (diversity OR “species diversity” OR phylogen* OR taxonomy)) AND ((coffea* OR “coffee*” OR “coffee cultivation”) OR (“*theobroma cacao*” OR “cacao” OR “cocoa”))
Mycoparasitism	(trichoderma* AND (mycoparasitic* OR “fungal parasit*” OR “parasitic interaction”))
Antibiosis	(trichoderma* AND (antibiosis OR “antimicrobial compound*” OR “secondary metabolite*”))
Nutrient and space competition	(trichoderma* AND (“nutrient competition” OR “space competition” OR “niche exclusion” OR “resource competition”))
Plant–microorganism interaction	(trichoderma* AND (“plant interaction” OR “root colonization” OR “rhizosphere”))
Plant growth promotion	(trichoderma* AND (“plant growth promot*” OR “growth enhancement” OR “root development”))
Induced systemic resistance	(trichoderma* AND (“induced resistance” OR “systemic resistance” OR “plant defense”))
Heavy-metal bioremediation	(trichoderma* AND (bioremediation OR “heavy metal*” OR cadmium OR “metal tolerance” OR “metal detoxification”))
Entomopathogenic potential	(trichoderma* AND (entomopathogen* OR “insect control” OR “insecticidal activity” OR “biocontrol of insects” OR “insect pathogenic fungi”))

#### 2.2.3 Inclusion and exclusion criteria

During the literature retrieval process in the Scopus and Web of Science databases, all articles published between 1985 and 2025 were considered, with particular emphasis on recent literature (2021–2025), since studies addressing mechanisms of action are continuously evolving. Both experimental studies and systematic reviews were included. Independent searches were performed for each previously identified mechanism of action, excluding subject areas not aligned with the scope of this study, such as zoology, occupational health, pharmacology, toxicology, and pharmaceutical sciences.

Bibliometric metadata were extracted from all relevant records, including author, title, source, year, and DOI. Data were exported in RIS format from both databases, and duplicates were removed using Zotero. In addition, studies without a clear description of *Trichoderma* mechanisms of action and literature not aligned with the study objectives were excluded through manual screening.

Titles and abstracts were independently evaluated by two of the authors to identify studies providing direct evidence of the mechanisms of action of *Trichoderma*. Any discrepancies were resolved by consensus with the participation of a third author, who reviewed the final selection to ensure the quality and relevance of the included literature. The selected documents were subsequently reviewed by all authors for validation and manuscript preparation. For each mechanism, up to 15 documents were considered, selected based on the following criteria: (i) thematic relevance, (ii) clarity in the description of the mechanism, and (iii) applicability to coffee and cacao agroecosystems.

## 3 Results and discussion

### 3.1 Bibliometric analysis

#### 3.1.1 Temporal evolution of scientific production and citation trends

**Figure 1:**
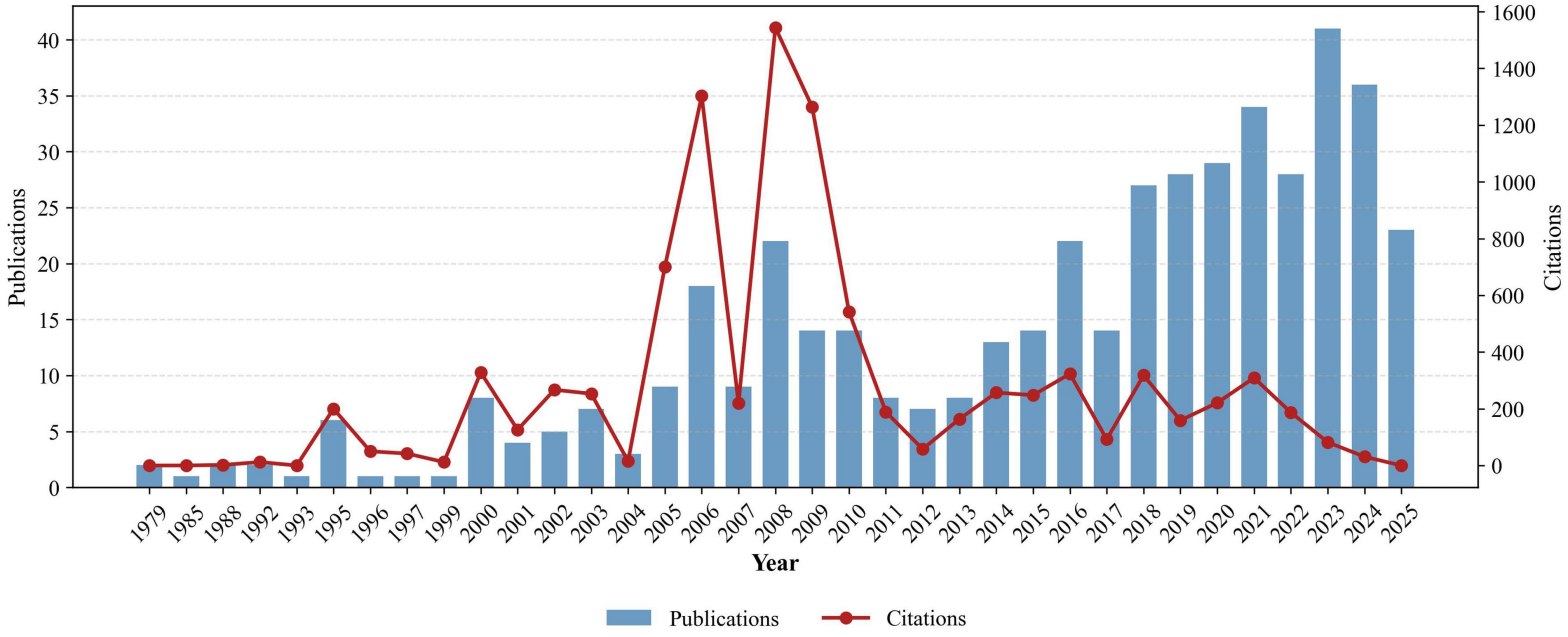
Temporal evolution of scientific production on *Trichoderma* in coffee and cacao agroecosystems between 1979 and mid-2025. Blue bars represent the annual number of publications, while the red line indicates the trend in annual citations as an indicator of academic impact.

Scientific production on *Trichoderma* in coffee and cacao agroecosystems has followed a progressive pattern over time (Figure 1). During the initial period (1979-1999), publications were limited and sporadic, with only 0–2 documents per year. From 2000 onwards, a steady and progressive increase was observed, with a marked acceleration after 2010. The highest peaks in publication output were recorded in 2023 and 2024, each exceeding 35 documents.

In contrast, citation patterns displayed a markedly different and more volatile trajectory than publication volume. Three peaks of high scientific impact were identified: 2006 (approximately 1,300 citations), 2008 (around 1,600 citations, the maximum recorded), and 2009 (approximately 1,250 citations). This period (2006–2009) represents the phase of greatest scientific influence in the field. After 2008, however, a sharp and sustained decline in annual citations was observed, stabilizing at considerably lower levels (generally below 400 citations per year) during 2009–2025. This trend reveals a disconnect between the growing productivity of publications and the declining scientific impact of research on *Trichoderma* in these agroecosystems.

#### 3.1.2 Geographic distribution of scientific production

**Figure 2:**
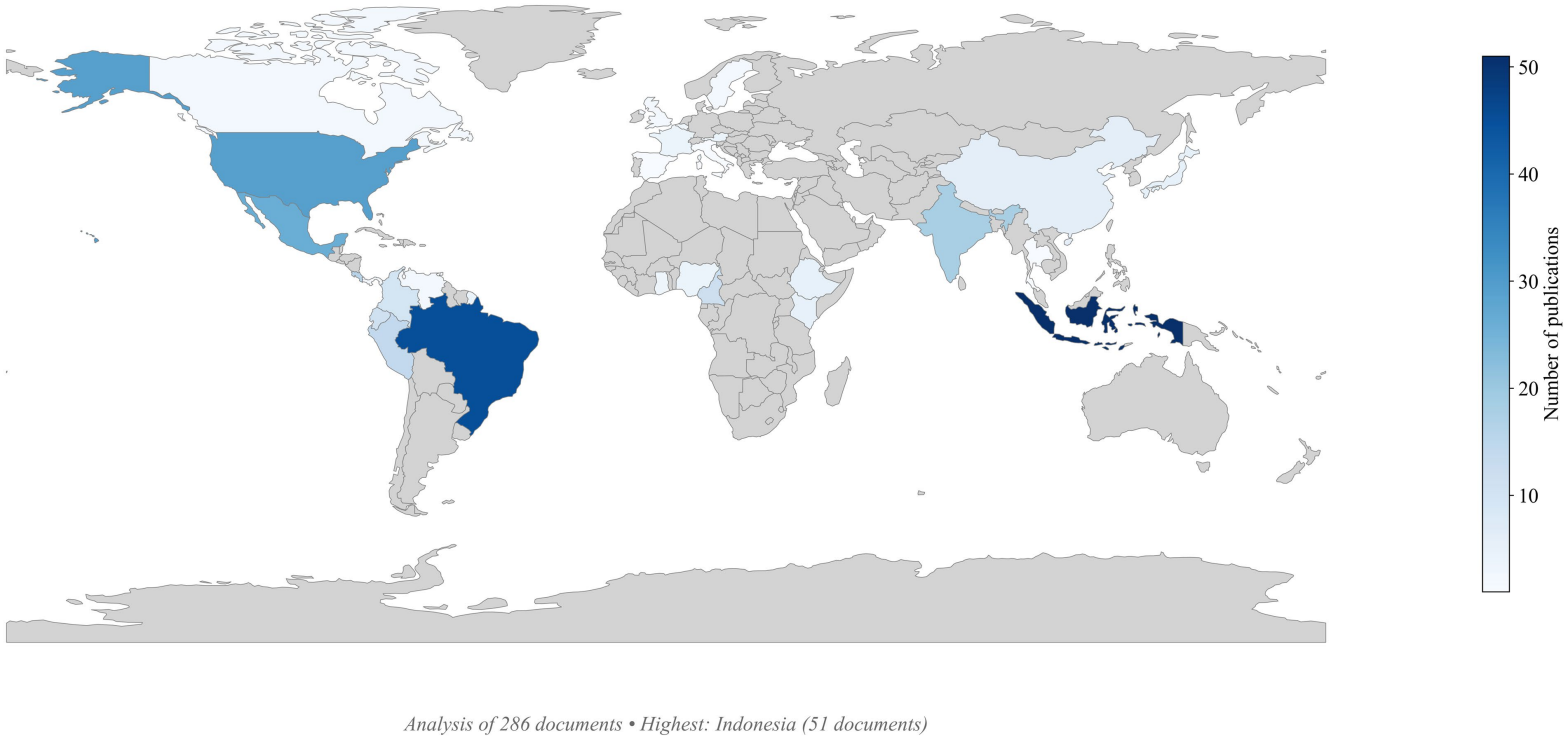
Geographic distribution of scientific production on *Trichoderma* in coffee and cacao agroecosystems worldwide.

A total of 286 occurrences (including co-publications) were detected, revealing a heterogeneous distribution with a clear concentration in tropical and subtropical regions where coffee and cacao are cultivated (Figure 2). Scientific production is led by Indonesia with 51 publications (17.8% of the global total), followed by Brazil with 45 documents (15.7%), the United States with 29 (10.1%), Mexico with 26 (9.1%), India with 18 (6.3%), Costa Rica with 16 (5.6%), Peru with 14 (4.9%), Cameroon with 12 (4.2%), Ecuador with 11 (3.8%), and Colombia with 10 (3.5%). Collectively, these top 10 countries account for 85.7% (245 documents) of the global output, underscoring that scientific knowledge production has consolidated within a relatively small group of countries.

At the regional level, Latin America emerges as the leading contributor with 45.4% of total publications. The Asia–Pacific region ranks second with 29.3%, followed by North America with 10.7% and Africa with 10.1%. Europe contributes the least, with only 6.6% of the global scientific production.

Keyword co-occurrence analysis and thematic patterns The keyword co-occurrence analysis revealed a total of 1,996 terms in the dataset, of which 240 reached the minimum threshold of five co-occurrences. The most relevant terms were “Trichoderma” (214 occurrences; total link strength = 2,218), followed by “biological control” (205 occurrences; total link strength = 2,027) and “*Theobroma cacao*” (203 occurrences; total link strength = 2,181). Additional prominent terms included “endophytes” (74 occurrences; link strength = 919) and “plant diseases” (32 occurrences; link strength = 533).

**Figure 3:**
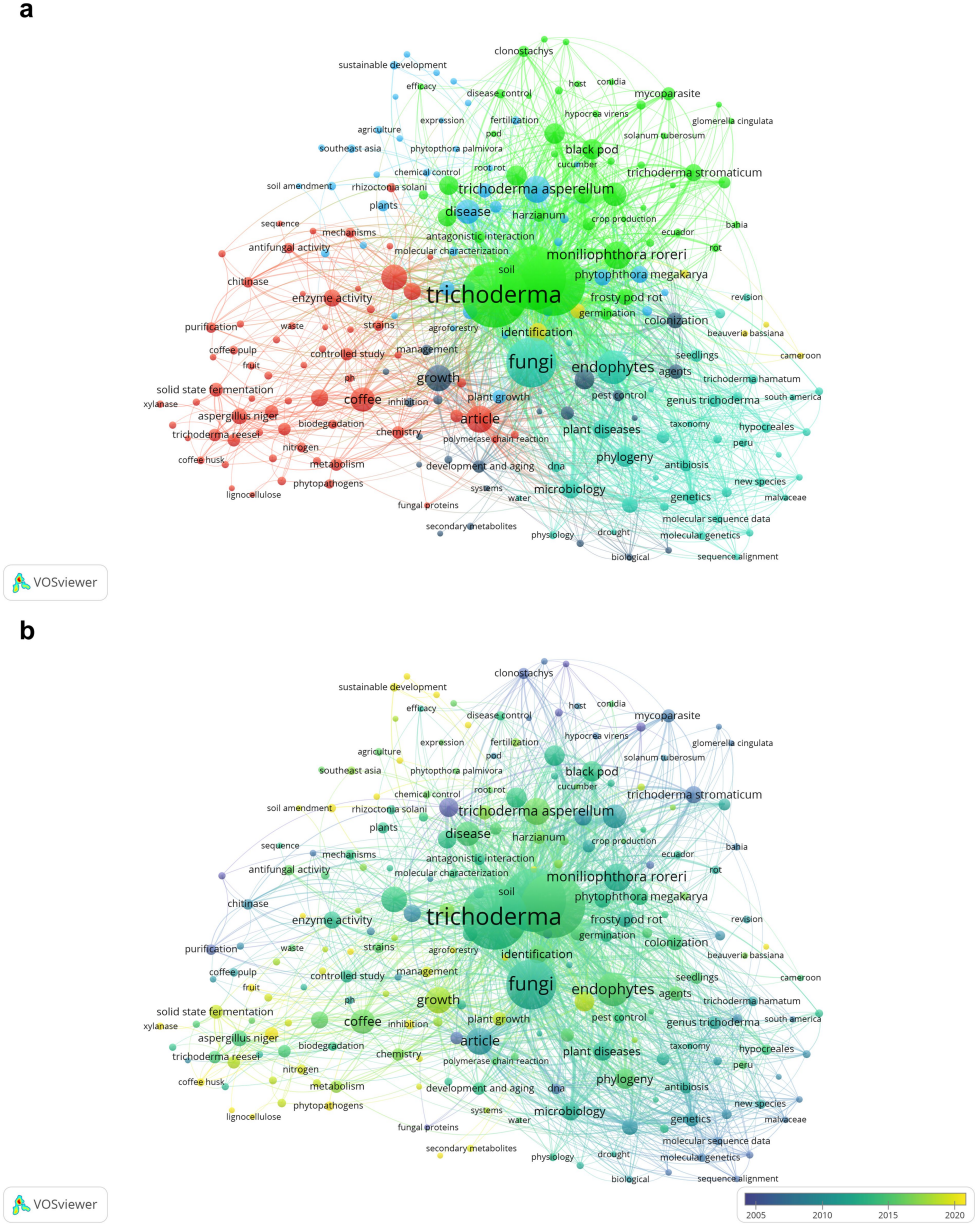
Conceptual structure of research on *Trichoderma* spp. in coffee and cacao agroecosystems. **(a)** Keyword co-occurrence map. **(b)** Temporal evolution of keywords.

The keyword visualization network was organized into six thematic clusters differentiated by color, each grouping terms based on semantic affinity and co-occurrence patterns (Figure 3). Cluster 1 (green) integrated concepts associated with *Trichoderma* spp., disease control, and endophytic fungi, highlighting linkages between biological control, fungal colonization, and pathogens such as *Moniliophthora roreri.* Cluster 2 (red) focused on biotechnological applications, particularly agricultural waste degradation, solid-state fermentation, lignocellulose, and enzymatic activity. Cluster 3 (blue) comprised terms related to plant growth and development, including “plant growth,” “fruit development,” and “cropping systems.” The remaining clusters reflected specialized areas: yellow for fungal taxonomy and phylogeny, light gray for molecular genetics, and purple for sustainable agricultural practices, illustrating the thematic diversity of research in this field.

The temporal overlay of keywords enabled identification of shifts in thematic focus across the literature, with node colors ranging from purple (earlier terms) to yellow (more recent terms). Keywords in blue tones, such as “taxonomy,” “antibiosis,” “phylogeny,” and “molecular genetics,” corresponded to earlier lines of research centered on fungal characterization and taxonomic foundations. In contrast, terms such as “*Trichoderma*,” “fungi,” “disease,” “endophytes,” and “*Moniliophthora roreri*” appeared in medium green, reflecting their sustained prominence in recent years and consolidation as core topics in the research network. Finally, concepts such as “solid-state fermentation,” “coffee pulp,” “biodegradation,” “metabolism,” and “lignocellulose” appeared in yellow, suggesting their emergence as novel themes strongly linked to applied biotechnology and waste valorization in sustainable production systems.

#### 3.1.3 Scientific impact analysis

The analysis of scientific impact highlights a group of highly influential researchers distinguished by their elevated citation counts, substantial average citations per publication, and outstanding H-index values, indicating that their contributions have exerted considerable influence in this field. This impact is primarily reflected in the number of citations received by their publications, which evidences the degree of recognition and relevance of their work within the scientific community.

Among them, G.J. Samuels emerges as the most influential author, with 27 publications, 2,787 citations (an average of 103 citations per document), and an H-index of 23. He is followed by A.W.V. Pomella, with 18 publications, 1,378 citations, and an H-index of 18, also reflecting substantial impact. Similarly, U. Krauss stands out with 16 publications, 680 citations, and an H-index of 16.

In terms of average impact per article, the results indicate that publication volume is not always directly correlated with total citation count. Some authors, despite producing fewer publications, have achieved high visibility and influence, as evidenced by their average citations per document and H-index (Table 2). For example, H. Bae has only eight publications, but with an average of 203 citations per document, his work has been widely recognized and frequently cited. Similarly, K.A. Holmes and M.D. Strem stand out with high averages of 140 and 104 citations per publication, respectively, despite a relatively modest number of documents. These findings underscore that research relevance in this field is defined less by productivity and more by the lasting impact of individual studies.

**Table 2:** Bibliometric indicators of leading researchers in *Trichoderma* studies in coffee and cacao agroecosystems.

Author	Publications	Total citations	Average citations	H-index
Samuels, GJ	27	2,787	103.22	23
Pomella, AWV	18	1,378	76.56	18
Krauss, U	16	680	42.50	16
Bailey, BA	16	1814	113.38	14
ten Hoopen, GM	15	512	34.13	14
Ismaiel, A	12	752	62.67	12
Strem, MD	12	1,684	140.33	12
Holmes, KA	10	1,042	104.20	10
Arroyo, C	10	302	30.20	10
Loguercio, LL	10	562	56.20	10
Begoude, BAD	9	455	50.56	9
de Souza, JT	10	182	18.20	8
Hebbar, PK	10	452	45.20	8
Hebbar, KP	8	574	71.75	8
Bae, H	8	1,630	203.75	8

Analysis of the ten most influential studies revealed that research has primarily focused on understanding the endophytic capacity of *Trichoderma* spp. species and the benefits of their interaction with host plants (Table 3). The most cited article corresponds to the study by Bae et al. (2009), *“The beneficial endophyte Trichoderma hamatum isolate DIS 219b promotes growth and delays the onset of the drought response in Theobroma cacao,”* published in Journal of Experimental Botany, with 375 citations. This work laid the foundation for understanding the role of *T. hamatum* as a beneficial endophyte, particularly in promoting plant growth and enhancing drought resistance in cacao.

**Table 3:** Most cited articles on *Trichoderma* spp. in coffee and cacao agroecosystems.

**Title**	**Year**	**Journal**	**Citations**	**Authors**	**Doi**
The beneficial endophyte *Trichoderma hamatum* isolate DIS 219b promotes growth and delays the onset of the drought response in *Theobroma cacao*	2009	*Journal of Experimental Botany*	375	[‘Bae, H’, ‘Sicher, RC’, ‘Kim, MS’, ‘Kim, SH’, ‘Strem, MD’, ‘Melnick, RL’, ‘Bailey, BA’]	10.1093/jxb/erp165
Endophytic fungi as biocontrol agents of *Theobroma cacao* pathogens	2008	*Biological Control*	362	[‘Mejia, LC’, ‘Rojas, EI’, ‘Maynard, Z’, ‘Van Bael, S’, ‘Arnold, AE’, ‘Hebbar, P’, ‘Samuels, GJ’, ‘Robbins, N’, ‘Herre, EA’]	10.1016/j.biocontrol.2008.01.012
Fungal and plant gene expression during the colonization of cacao seedlings by endophytic isolates of four *Trichoderma* species	2006	*Planta*	221	[‘Bailey, BA’, ‘Bae, H’, ‘Strem, MD’, ‘Roberts, DP’, ‘Thomas, SE’, ‘Crozier, J’, ‘Samuels, GJ’, ‘Choi, IY’, ‘Holmes, KA’]	10.1007/s00425-006-0314-0
Diversity of endophytic fungal community of cacao (*Theobroma cacao* L.) and biological control of *Crinipellis perniciosa,* causal agent of Witches’ Broom Disease	2005	*International Journal of Biological Sciences*	220	[‘Rubini, MR’, ‘Silva-Ribeiro, RT’, ‘Pomella, AWV’, ‘Maki, CS’, ‘Araújo, WL’, ‘dos Santos, DR’, ‘Azevedo, JL’]	10.7150/ijbs.1.24
The *Trichoderma koningii* aggregate species	2006	*Studies in Mycology*	185	[‘Samuels, GJ’, ‘Dodd, S’, ‘Lu, BS’, ‘Petrini, O’, ‘Schroers, HJ’, ‘Druzhinina, IS’]	10.3114/sim.2006.56.03
Antibiosis, mycoparasitism, and colonization success for endophytic *Trichoderma* isolates with biological control potential in *Theobroma cacao*	2008	*Biological Control*	154	[‘Bailey, BA’, ‘Bae, H’, ‘Strem, MD’, ‘Crozier, J’, ‘Thomas, SE’, ‘Samuels, GJ’, ‘Vinyard, BT’, ‘Holmes, KA’]	10.1016/j.biocontrol.2008.01.003
*Hypocrea rufa/Trichoderma viride:* a reassessment, and description of five closely related species with and without warted conidia	2006	*Studies in Mycology*	137	[‘Jaklitsch, WM’, ‘Samuels, GJ’, ‘Dodd, SL’, ‘Lu, BS’, ‘Druzhinina, IS’]	10.3114/sim.2006.56.04
Endophytic fungal diversity in *Theobroma cacao* (cacao) and *T. grandiflorum* (cupuacu) trees and their potential for growth promotion and biocontrol of black-pod disease	2010	*Fungal Biology*	134	[‘Hanada, RE’, ‘Pomella, AWV’, ‘Costa, HS’, ‘Bezerra, JL’, ‘Loguercio, LL’, ‘Pereira, JO’]	10.1016/j.funbio.2010.08.006
Long-Term Coffee Monoculture Alters Soil Chemical Properties and Microbial Communities	2018	*Scientific Reports*	127	[‘Zhao, QY’, ‘Xiong, W’, ‘Xing, YZ’, ‘Sun, Y’, ‘Lin, XJ’, ‘Dong, YP’]	10.1038/s41598-018-24537-2
Molecular characterization and antimicrobial activity of endophytic fungi from coffee plants	2006	*World Journal of Microbiology & Biotechnology*	121	[‘Sette, LD’, ‘Passarini, MRZ’, ‘Delarmelina, C’, ‘Salati, F’, ‘Duarte, MCT’]	10.1007/s11274-006-9160-2

Similarly, studies on biological control of pathogens have been pivotal, such as the article by Mejia et al. (2008) in *Biological Control* (362 citations), which established the role of endophytic fungi, including *Trichoderma* spp., in protecting cacao against diseases. Other relevant contributions include investigations on fungal diversity and taxonomy; host–fungus gene expression during *Trichoderma* colonization (Bailey et al., 2006, Planta, 221 citations); and the diversity of endophytic fungi and their capacity to control *Crinipellis perniciosa,* the causal agent of witches’ broom disease (Rubini et al., 2005, 220 citations). More recent studies, such as Zhao et al. (2018) in *Scientific Reports* (127 citations), reflect an evolution toward ecological and microbiome-focused perspectives in cacao systems.

Of the total studies analyzed, 80% focused on *Theobroma cacao* and only 20% on *Coffea arabica*, indicating a marked concentration of research on cacao within the context of studying the endophytic capacity of *Trichoderma* spp. The predominance of journals such as *Biological Control* and *Studies in Mycology* further underscores the applied and taxonomic nature of this research field, integrating fungal ecology, biotechnology, and sustainable disease management.

### 3.2 Systematic analysis: ecology and functions of *Trichoderma* spp

#### 3.2.1 Ecological and functional diversity in coffee and cacao agroecosystems

The systematic analysis of the literature revealed a wide distribution and remarkable diversity of *Trichoderma* in tropical agroecosystems. At least 39 species have been reported in coffee and cacao production systems, underscoring their ecological versatility through both their common presence in the rhizosphere and their ability to establish epiphytic and endophytic associations with both crops (Bailey et al., 2009; Torres-De la Cruz et al., 2015; Lu et al., 2022; Waqar et al., 2024).

Of the total species recorded, 24 were reported to colonize both crops, while 11 were specific to coffee and 4 were associated exclusively with cacao. Regarding sources of isolation, the rhizosphere was the most frequent site, with 23 species identified, including *T. viride, T. asperellum,* and *T. harzianum,* all recognized for their biocontrol potential. Additionally, 18 species were isolated from stems and internal tissues, indicating an endophytic lifestyle. Other species were recovered from dead branches, suggesting a saprophytic or decomposer role, as observed in *T. andinense* and *T. orientale.*

Among the most frequently reported species were *T. harzianum, T. hamatum, T. spirale, T. asperelloides, T. asperellum,* and *T. longibrachiatum* (Sánchez Hernández et al., 2018; Mulatu et al., 2022). Of these, *T. harzianum* stands out as one of the most extensively studied species due to its strong biocontrol potential and notable adaptability to colonize multiple ecological niches, making it a key species for the biological management of diseases in tropical crops.

In functional terms, several species with demonstrated biocontrol capacity were identified, including *T. aethiopicum, T. asperellum, T. atroviride, T. virens, T. stromaticum,* and *T. theobromicola.* These species have shown efficacy in suppressing major phytopathogens of economic relevance such as *Moniliophthora perniciosa, M. roreri, Phytophthora palmivora,* and *Hemileia vastatrix*. Furthermore, several endophytic species - such as *T. botryosum, T. ovalisporum, T. spirale,* and *T. martiale* - have been identified as potential inducers of systemic resistance mechanisms in host plants, thereby contributing to improved plant health (Supplementary Table S2).

#### 3.2.2 Mechanisms of action in microbe-plant interactions

##### 3.2.2.1 Plant colonization process

**Figure 4:**
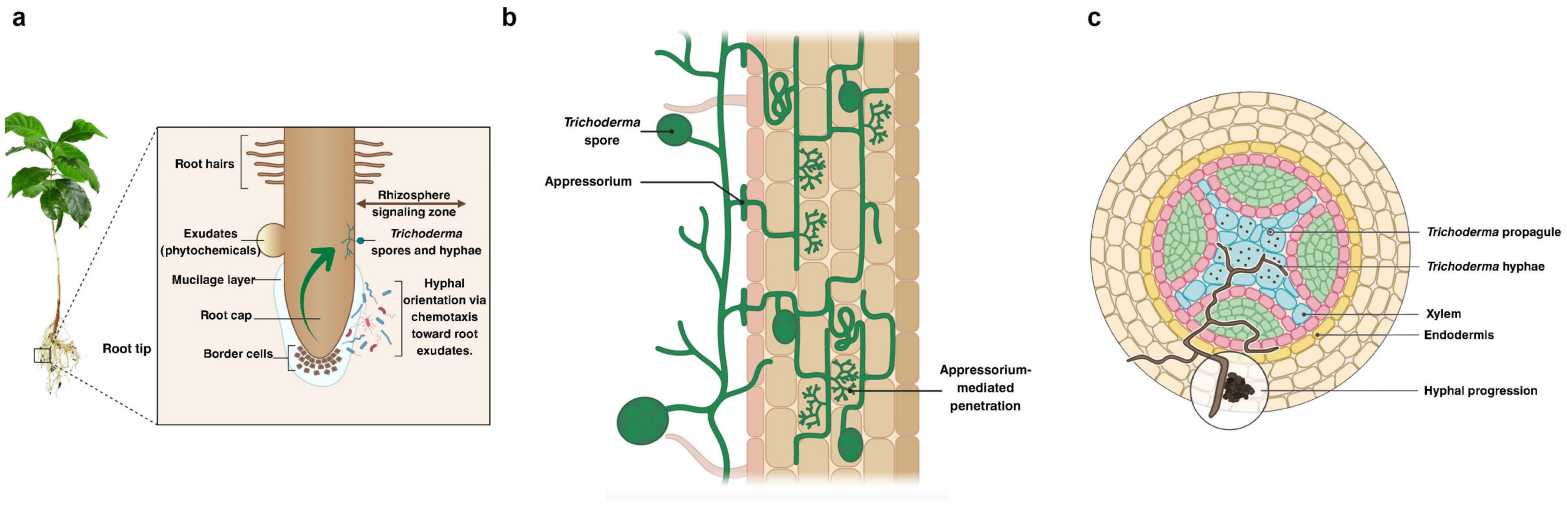
Colonization of plant roots by *Trichoderma.* **(a)** Recognition and chemotaxis in the rhizosphere: *Trichoderma* spores and hyphae orient toward root surfaces in response to exuded phytochemicals. **(b)** Adhesion and penetration: formation of appressoria and hyphal invasion into epidermal and cortical cells. **(c)** Endophytic colonization and systemic progression: hyphae and propagules colonize the root cortex and xylem, enabling systemic migration within host tissues. This figure was created with BioRender.com and Canva.

The interaction of *Trichoderma* with plants involves a complex sequence of mechanisms leading to the colonization of internal plant tissues and the activation of defense systems against both biotic and abiotic stress (Dutta et al., 2023) (Figure 4). The process begins when the microorganism perceives phytochemical signals present in root exudates - such as sugars, flavonoids, amino acids, organic acids, and strigolactones - whose production is often enhanced under stress conditions (Guzmán-Guzmán et al., 2025; Yang et al., 2025) (Figure 4a).

In response to these stimuli, *Trichoderma* establishes initial contact with the rhizosphere and adheres to root surfaces through specific adhesins, including hydrophobins and ceratoplatanins (Dutta et al., 2023). Subsequently, *Trichoderma* employs proteins such as swollenin, together with a suite of cell wall–degrading enzymes (CWDEs), including xylanases, endopolygalacturonases, cellulases, and pectinases. These enzymes facilitate the controlled penetration of root tissues without causing structural damage. In addition to promoting colonization, CWDEs release compounds associated with damage-associated molecular patterns (DAMPs), which function as signaling molecules in plant–microbe communication. Specifically, *Trichoderma* has been shown to release oligo-galacturonides and chitooligosaccharides derived from enzymatic activity on endogenous polysaccharides, which trigger the activation of defense pathways in roots. Thus, CWDEs not only partially soften the cell wall and alter its plasticity but also promote apoplastic colonization and the establishment of a symbiotic interaction between *Trichoderma* and the host plant (Yang et al., 2025) (Figure 4b). Under favorable conditions, some strains can extend beyond the root cortex, reaching the xylem and migrating systemically into stems and leaves (endosphere), and in some cases, even colonizing the phyllosphere (leaf surface) (Figure 4c).

This capacity depends largely on the metabolic and genetic adaptability of fungal strains to tolerate plant defense metabolites such as phenols and terpenoids (Guzmán-Guzmán et al., 2025). Endophytic strains typically display a greater diversity of biosynthetic gene clusters (BGCs) and degradative gene clusters (DGCs), which are associated with intracellular adaptation and the evasion of host defense responses (Guzmán-Guzmán et al., 2025; Yang et al., 2025).

##### 3.2.2.2 Induction of systemic resistance

Once the interaction is established, *Trichoderma* activates the plant immune system through two complementary mechanisms. The first corresponds to pattern- or microbe-triggered immunity (PTI/MTI), in which plants recognize MAMPs (microbe-associated molecular patterns), such as chitin, β-glucans, and oligogalacturonides, through pattern-recognition receptors (PRRs). This initial recognition induces early defense responses characterized by ion fluxes across the plasma membrane, activation of mitogen-activated protein kinases (MAPKs), accumulation of reactive oxygen species (ROS), and reinforcement of the cell wall through callose deposition (Tyśkiewicz et al., 2022; Dutta et al., 2023).

The second mechanism, effector-triggered immunity (ETI), involves the recognition of fungal effectors by intracellular resistance proteins (R-proteins), leading to a localized hypersensitive response (HR) (Yang et al., 2025; Hermosa et al., 2012).

Both mechanisms converge into a complex signaling network that regulates systemic defense responses, including the synthesis of phytoalexins, pathogenesis-related (PR) proteins, and secondary metabolites (Tyśkiewicz et al., 2022; Guzmán-Guzmán et al., 2025). Depending on the hormonal balance and the nature of the interaction, these pathways may lead to different types of induced resistance: systemic acquired resistance (SAR), mediated by salicylic acid (SA), and induced systemic resistance (ISR), dependent on jasmonic acid (JA) and ethylene (ET) (Tyśkiewicz et al., 2022; Yang et al., 2025).

During *Trichoderma* colonization, various elicitors such as cell wall fragments, peptides, volatile organic compounds, and siderophores modulate the JA and ET signaling pathways, activating ISR without causing tissue damage. This type of resistance induces a “defense priming” state, enabling plants to respond faster and more robustly to subsequent infections (Dutta et al., 2023; Guzmán-Guzmán et al., 2025).

ROS play a dual role in these processes. At moderate concentrations, they act as essential second messengers in defense signaling, participating in MAPK activation, hormone crosstalk, and the expression of genes associated with ISR (Villalobos-Escobedo et al., 2020; Dutta et al., 2023). However, excessive accumulation of ROS leads to oxidative stress and programmed cell death, characteristics of SAR and the hypersensitive response (HR) (Yang et al., 2025).

Therefore, ISR induced by *Trichoderma* relies on precise control of the redox balance: moderate oxidative bursts function as defense signals, whereas antioxidant systems such as peroxidases, catalases, and superoxide dismutases mitigate cellular damage and maintain symbiotic compatibility between the fungus and the plant (Hermosa et al., 2012; Guzmán-Guzmán et al., 2025).

Thus, *Trichoderma* modulates both local and systemic plant immunity through hormonal and redox-dependent signaling cascades, integrating MAPK phosphorylation, ROS regulation, and phytohormone crosstalk into an efficient defense network that enhances plant resilience against biotic and abiotic stresses (Tyśkiewicz et al., 2022; Dutta et al., 2023; Guzmán-Guzmán et al., 2025).

##### 3.2.2.3 Plant growth promotion

In addition to modulating plant immunity, *Trichoderma* exhibits a remarkable ability to promote plant growth through hormonal, nutritional, and metabolic mechanisms. At the hormonal level, strains such as *T. atroviride* and *T. virens* harbor biosynthetic gene pathways for the production of auxins (IAA), gibberellins (GA), cytokinins (CK), abscisic acid (ABA), and ethylene (ET) (Dutta et al., 2023; Guzmán-Guzmán et al., 2025). These compounds are secreted into the rhizosphere or endosphere, where they act on plant cells to promote root elongation and branching (IAA), stimulate cell division and leaf maturation (GA, CK), and enhance stress tolerance (ABA, ACC deaminase activity) (Dutta et al., 2023; Guzmán-Guzmán et al., 2025).

*Trichoderma* also improves nutrient bioavailability by enhancing the uptake of phosphorus, iron, zinc, and manganese through the production of siderophores, organic acids, and mineralizing enzymes (Guzmán-Guzmán et al., 2025). This activity significantly contributes to plant nutrition and productivity, particularly in nutrient-poor or degraded soils (Dutta et al., 2023). Moreover, the secretion of volatile compounds such as 6-pentyl-*α*-pyrone (6-PP) has been shown to directly stimulate root development, photosynthetic gene expression, and aerial biomass accumulation, ultimately leading to improved crop yield (Dutta et al., 2023; Guzmán-Guzmán et al., 2025).

#### 3.2.3 Biocontrol mechanisms against phytopathogens

##### 3.2.3.1 Mycoparasitism

**Figure 5:**
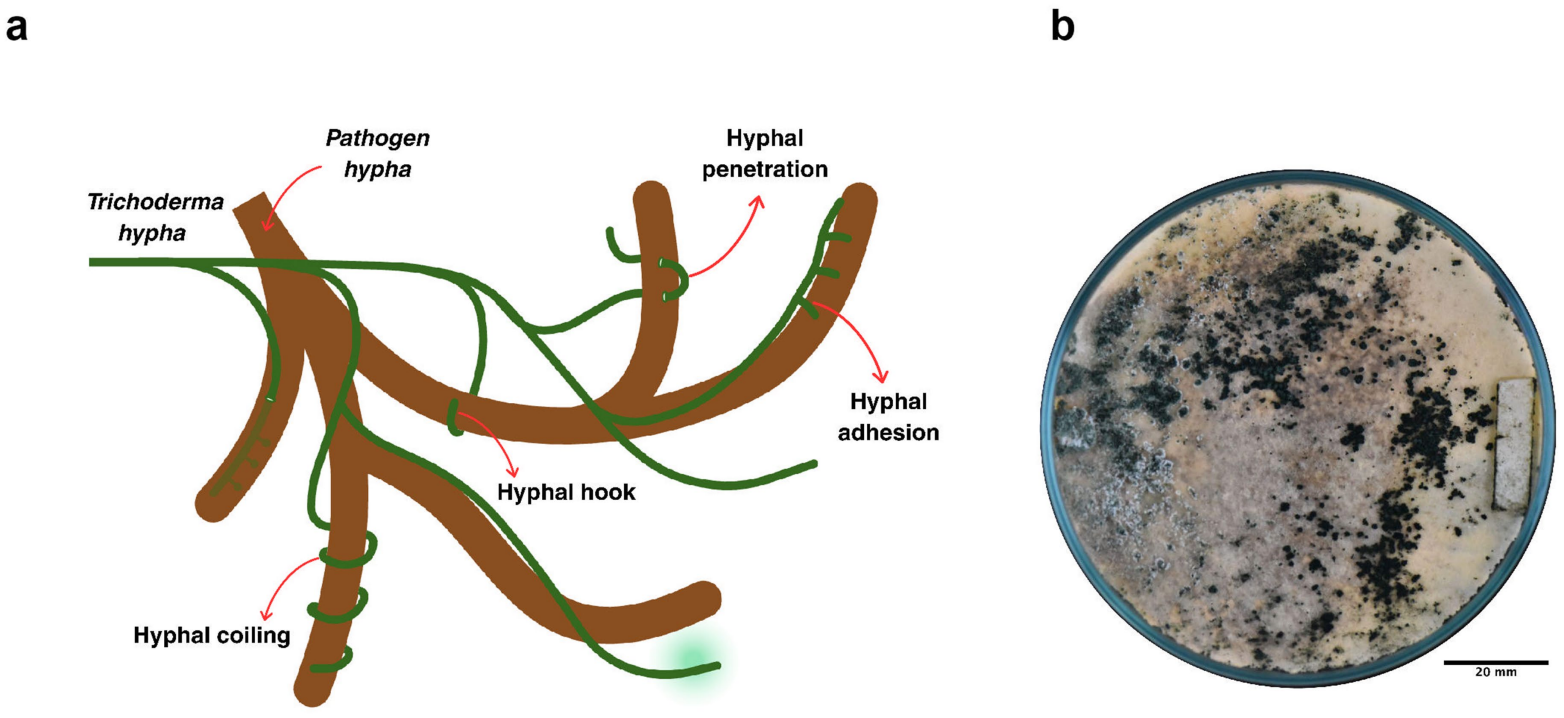
Mycoparasitic interaction of *Trichoderma* with phytopathogenic fungi. **(a)** Diagrammatic representation of the mycoparasitic process, illustrating sequential events including hyphal coiling, hook formation, adhesion, and penetration of *Trichoderma* hyphae into pathogen hyphae. **(b)** Evidence of *Trichoderma* sp. antagonism against *Moniliophthora roreri*, illustrating overgrowth and colonization of the pathogen culture under *in vitro* conditions. This figure was created with BioRender.com and Canva.

The antagonistic activity of *Trichoderma* species against phytopathogens is mediated through a combination of direct and indirect mechanisms (Figure 5). During direct antagonism, *Trichoderma* produces a synergistic blend of bioactive compounds that act effectively in biological control (Chatterton and Punja, 2009; Asad, 2022). Among these strategies, mycoparasitism is a hallmark behavior of the genus and involves a complex sequence of events (Mukherjee et al., 2022).

The process begins when the fungus recognizes specific molecules released by the phytopathogen, triggering hyphal attack and coiling around the host hyphae (Figure 5a). At this stage, *Trichoderma* secretes enzymes that degrade the pathogen’s cell wall, allowing penetration and subsequent parasitism of host hyphae (Yang, 2017; Sánchez Hernández et al., 2018; Asad, 2022).

In the active phase of mycoparasitism, the interaction is reinforced by the binding of host cell wall carbohydrates to lectins of the pathogen (Asad, 2022). This interaction accelerates the invasion of *Trichoderma* hyphae into the host through penetration structures and cavities, supported by the secretion of antibiotics and cell wall-degrading enzymes during hyphal coiling (Sánchez Hernández et al., 2018) (Figure 5b).

Mycoparasitism is regulated by approximately 30 genes, the production of secondary metabolites, and the secretion of hydrolytic enzymes. Key among these are chitinases, glucanases, and proteases, which act in a coordinated manner to degrade the host’s cell wall. Endochitinases, which are abundant in *Trichoderma* genomes, initiate the attack by cleaving β-1,4 linkages in structural chitin (Asad, 2022; Mukherjee et al., 2022). Complementary CWDEs, including β-1,3-glucanases, β-1,6-glucanases, and mannosidases, subsequently hydrolyze remaining polymers, culminating in the complete lysis of the pathogen’s cell wall (Asad, 2022).

Comprehensive genomic and transcriptomic analyses have substantially deepened the understanding of this mechanism. Research conducted by Mukherjee and collaborators demonstrated that mycoparasitism represents an ancestral and evolutionarily conserved trait within the genus *Trichoderma*, supported by the expansion of gene families associated with cell wall degradation, secondary metabolism, and signal transduction (Mukherjee et al., 2012; Mukherjee et al., 2022). During confrontation with phytopathogenic fungi, *Trichoderma* overexpresses genes encoding glycoside hydrolases such as GH18 chitinases, GH55 β-1,3-glucanases, and S8 subtilisin-like proteases that act synergistically to lyse the host cell wall, while secondary metabolites, including peptaibols and gliotoxin, reinforce the attack process (Mukherjee et al., 2013; Mukherjee et al., 2022). Furthermore, it has been proposed that *Trichoderma* mycoparasitism may shift from a necrotrophic to a hemibiotrophic phase, allowing transient intracellular colonization of the host fungus prior to its complete degradation, thereby enhancing the physiological plasticity and ecological adaptability of the genus (Mukherjee et al., 2022). Collectively, these findings indicate that integrated regulatory networks including G protein-coupled receptors, MAP kinase cascades (Tmk1/Tmk3), and cAMP-mediated (cyclic adenosine monophosphate) signaling pathways coordinate gene expression involved in host recognition, hyphal coiling, secretion of hydrolytic enzymes, and the biosynthesis of secondary metabolites (Mukherjee et al., 2012; Mukherjee et al., 2022).

##### 3.2.3.2 Antibiosis

Secondary metabolites play a crucial role in direct antagonism against pathogens while simultaneously modulating the composition and functionality of the root-associated microbiome. Among these compounds, peptaibols act not only as potent antimicrobial agents but also promote the establishment of beneficial associations in the rhizosphere by displacing harmful microorganisms. Similarly, butenolides have been highlighted for their remarkable antibiotic activity against the development of phytopathogens (Asad, 2022). In addition to their direct antagonistic effects, these compounds stimulate plant defense responses, thereby improving crop health and promoting more sustainable agricultural production (Reino et al., 2007).

Although many *Trichoderma*-derived secondary metabolites remain incompletely characterized, available evidence suggests that they play key roles in the ecological adaptation and competitive survival of the genus across diverse environments (Zeilinger et al., 2016). Recent genomic and transcriptomic studies have revealed the presence of multiple gene families involved in the biosynthesis of antibiotic compounds, including polyketide synthases (PKS), nonribosomal peptide synthetases (NRPS), and enzymes associated with the formation of terpenoids and volatile compounds (Mukherjee et al., 2011, 2013, 2022). The expression of these metabolic clusters is regulated by Velvet-type protein complexes and intracellular signaling pathways mediated by MAP kinases and cyclic adenosine monophosphate (cAMP), which coordinate metabolite production in response to environmental cues and the presence of competing fungi (Mukherjee et al., 2022).

Among the most representative metabolites of *Trichoderma* are peptaibols, epipolythiodioxopiperazines such as gliotoxin, and volatile compounds like 6-pentyl-2H-pyran-2-one (6-PP), which act synergistically with hydrolytic enzymes during mycoparasitism. These molecules exert fungistatic and fungicidal effects against agriculturally important pathogens by altering membrane permeability and compromising the physiological integrity of host hyphae (Mukherjee et al., 2012; Mukherjee et al., 2022). Moreover, 6-PP has been shown to perform a dual role: as an antifungal metabolite and as a signaling molecule that stimulates *Trichoderma* mycelial growth and directs chemotaxis toward the substrate or host.

Additionally, siderophores produced by various *Trichoderma* species contribute to iron competition within the rhizosphere, limiting its availability to pathogenic microorganisms and enhancing the effectiveness of biological control (Mukherjee et al., 2022). A summary of the main secondary metabolites and microbial volatile organic compounds (mVOCs) reported in Trichoderma species, along with their biological activities, is provided in Table 4.

**Table 4:** Secondary metabolites and microbial volatile organic compounds (mVOCs) produced by *Trichoderma* species and their biological activity.

Type of compound	Compound name	Producing species	Biological activity	DOI/Reference
Secondary metabolite	Gliotoxin	*T. virens*, *T. harzianum*	Antifungal	10.1038/s41598-021-95907-6; 10.3389/fagro.2022.932839
	Viridin	*T. virens*	Antifungal	10.1007/978-981-13-5862-3_5
	Gliovirin	*T. virens*	Antifungal	10.1007/978-981-13-5862-3_5; 10.1002/slct.201700262
	Heptelidic acid	*T. virens*	Antimicrobial	10.1007/978-981-13-5862-3_5
	Isoharziandione	*T. harzianum*, *T. viride*	Antifungal	10.1007/978-981-13-5862-3_5
	Harzialactone A/B	*T. harzianum*	Antimicrobial	10.1007/978-981-13-5862-3_5
	Trichodermamides A/B	*T. virens*, *T. harzianum*, *T. asperellum*	Cytotoxic, antifungal	10.1007/978-981-13-5862-3_5; 10.3389/fagro.2022.932839
	Peptaibols	*T. longibrachiatum*, *T. harzianum*, *T. virens*, *T. brevicompactum*, *T. viride*	Antifungal, membrane-disrupting	10.3390/plants9060762; 10.3389/fagro.2022.932839; 10.1007/978-981-13-5862-3_5; 10.1016/j.fbr.2016.05.001
	Polyketides	*T. atroviride*, *T. virens*	Antifungal	10.3389/fagro.2022.932839; 1016/j.fbr.2016.05.001; 10.1007/978-981-13-5862-3_5
	Koninginins	*T. koningii*	Antifungal	10.3389/fagro.2022.932839; 10.1007/978-981-13-5862-3_5
	Viridepyronone	*T. viride*	Antifungal	10.1007/978-981-13-5862-3_5
	Viridenepoxydiol	*T. viride*	Antifungal	10.1007/978-981-13-5862-3_5
mVOC	6-Pentyl-α-pyrone	*T. atroviride*, *T. asperellum*, *T. harzianum*, *T. hamatum*, *T. viride*, *T. koningii*	Antifungal, plant growth-promoting	10.3390/biology10090897; 10.1007/978-981-13-5862-3_5
	Limonene	*T. atroviride*	Growth-promoting	10.1007/978-981-13-5862-3_5
	α-Farnesene	*T. virens*	Antifungal, signaling	10.1007/978-981-13-5862-3_5; 10.1016/j.micres.2020.126552
	β-Bisabolene	*T. asperellum*, *T. virens*	Antifungal	10.1007/978-981-13-5862-3_5
	Farnesol	*T. virens*	Antifungal	10.1007/978-981-13-5862-3_5
	β-Elemene	*T. virens*, *T. asperellum*	Plant growth-promoting	10.1007/978-981-13-5862-3_6
	1-Octen-3-ol	*T. harzianum*, *T. asperellum*	Developmental signaling	10.1007/978-981-13-5862-3_7
	3-Octanone / 3-Octanol	*T. harzianum*, *T. asperellum*	Developmental signaling	10.1007/978-981-13-5862-3_8
	Benzoic acid derivatives	*T. harzianum*, *T. viride*	Antimicrobial	10.1007/978-981-13-5862-3_9
	Dimethyl disulfide/Methanethiol	*T. gamsii*	Antifungal	10.1007/978-981-13-5862-3_10

##### 3.2.3.3 Competitive exclusion of phytopathogens by *Trichoderma*

*Trichoderma* is recognized as an aggressive root colonizer with a strong capacity to compete for space and essential resources such as nutrients, water, and oxygen in the rhizosphere. This fungus mobilizes otherwise immobile soil nutrients, effectively displacing other microorganisms that inhabit the same niche (Dutta et al., 2023). Competition in the rhizosphere is further intensified by the diversified composition of root exudates, which represent critical resource hotspots that *Trichoderma* exploits through its metabolic versatility and rapid colonization ability (Yang et al., 2025). This competitive aggressiveness is also associated with the production of diverse secondary metabolites with antagonistic effects on cohabiting microorganisms (Dutta et al., 2023; Guzmán-Guzmán et al., 2025).

One of the key mechanisms underlying nutritional competition is the production of siderophores - iron-chelating molecules that sequester this essential micronutrient, thereby restricting its availability to other fungi and reducing their viability (Dutta et al., 2023). This strategy is particularly relevant in the biocontrol of soilborne pathogens whose development depends on iron availability (Contreras-Cornejo et al., 2016; Dutta et al., 2023). Additionally, the rapid growth of *Trichoderma* spp., combined with its hyphal confluence around roots and the production of antibiotic peptides such as those from *T. virens* Gv.29–8, enables the displacement of major pathogens, including *Fusarium* spp.*, Phytophthora* sp., *Rhizoctonia* sp., and *Sclerotium rolfsii* (Contreras-Cornejo et al., 2024).

This competitive activity can promote sporulation and dispersal of *Trichoderma,* reinforcing its persistence in the rhizosphere ecosystem (Contreras-Cornejo et al., 2024). Furthermore, its high growth rates are associated with the rapid utilization of carbon sources such as glucose and sucrose, supported by high-affinity glucose transporters that confer a competitive advantage in nutrient-poor environments (Tyśkiewicz et al., 2022; Dutta et al., 2023). In addition, the secretion of organic acids such as gluconic, citric, and fumaric acid contributes to the solubilization of phosphates and micronutrients (Fe, Mn, Mg), thereby improving nutrient availability for the host plant while simultaneously restricting access for competing microorganisms (Vinale et al., 2006; Tyśkiewicz et al., 2022).

##### 3.2.3.4 Research gaps and frontiers of knowledge

The systematic mapping of knowledge gaps in the literature revealed several underexplored thematic areas. A cluster of terms associated with sustainable agriculture and waste management - such as *“agro-industrial waste,” “humic acids,” “organic coffee,”* and *“biochar”*—was identified. Although these concepts appeared in earlier studies, they remain weakly represented and poorly integrated within the broader thematic network, suggesting that these research lines have received limited attention over the past decades.

A second critical gap relates to underrepresented pests and plant diseases, particularly those affecting tropical crops such as coffee and cacao. Terms including *“rot disease,” “Fusarium* sp.*,” “damping off,”* and “*Lasiodiplodia”* emerged only marginally in the network, despite the fact that these pathogens pose persistent threats from early developmental stages, significantly constraining yield and quality of export-oriented commodities.

Additionally, the role of *Trichoderma* in mycorrhizal interactions and its potential application in bioremediation processes remains scarcely addressed. The marginal occurrence of terms such as *“bioremediation”* and *“cadmium”* highlights the limited exploration of this fungus as a candidate for the remediation of metal-contaminated soils, despite its promising implications for both environmental sustainability and agricultural productivity.

Collectively, these gaps underscore the need for interdisciplinary research lines that integrate microbial biotechnology, sustainable agriculture, and environmental management. Bridging these frontiers may position *Trichoderma* spp. not only as a biocontrol agent but also as a cornerstone of agroecological innovation in tropical cropping systems.

### 3.3 Discussion

#### 3.3.1 Historical evolution of research

An interpretation of the observed thematic distribution reveals marked differences between the crops studied. The predominance of research focused on *Theobroma cacao* over *Coffea arabica* can be attributed to historical, economic, and phytopathological factors. The interaction between *Trichoderma* and cacao has been investigated for several decades due to the severe losses caused by pathogens such as *Moniliophthora perniciosa* and *Phytophthora palmivora*, which prompted an early and sustained search for endophytic agents with biocontrol potential. In contrast, studies on endophytic associations in coffee have gained relevance only in recent years, as research on this crop has traditionally emphasized rhizospheric and mycorrhizal interactions rather than its internal microbiota. Furthermore, cacao has served as a perennial model system for studying the functional genomics and ecology of *Trichoderma* spp., leading to a greater number of high-impact publications and, consequently, higher visibility and citation rates compared with coffee-focused research.

The earliest studies on *Trichoderma* in coffee and cacao agroecosystems were scarce and largely exploratory, consistent with the limited number of publications recorded between 1979 and 1999 (Figure 1). The pioneering work of Brasier and Griffin (1979) investigated the influence of *Trichoderma* species on the formation of sexual structures in *Phytophthora,* contributing to an improved understanding of pathogen reproductive biology. This study also suggested potential antagonistic roles of *Trichoderma* in shaping *Phytophthora* population dynamics in cacao plantations. Although these insights provided a conceptual foundation for biocontrol, they did not yet generate measurable bibliometric impact.

A few years later, Tauk (1985) demonstrated that *Trichoderma viride* significantly accelerated the decomposition of coffee pulp, enabling its use as an organic amendment for rehabilitating degraded soils in coffee plantations. This breakthrough opened a research avenue linking *Trichoderma* to coffee by-products. Follow-up studies confirmed its increased proliferation in the presence of coffee residues (Onsando and Waudo, 1992) and further revealed its ability to degrade caffeine (Roussos et al., 1995), positioning the fungus as a promising biotechnological agent for agro-industrial waste valorization.

From the early 2000s onwards, a clear upward trajectory in scientific output emerged (Figure 1), reflecting a global shift towards sustainable alternatives to agrochemicals. Research emphasis moved toward the *Trichoderma–plant–pathogen* interface, where bioactive compounds were characterized for their multifunctional roles in plant protection (de Azevedo et al., 2000; De Marco et al., 2000). In cacao, this period highlighted hydrolytic enzymes such as chitinases, glucanases, and proteases as key mediators of antagonism against *Moniliophthora roreri, M. perniciosa,* and *Phytophthora* spp. (De Marco et al., 2000; De Marco and Felix, 2002). These findings were validated across multiple laboratory and field studies (Krauss and Soberanis, 2001, 2002; García et al., 2003), reinforcing the robustness of enzymatic biocontrol as a research domain. Concurrently, several studies reported the endophytic colonization of *Theobroma cacao* by *Trichoderma* species, expanding the functional spectrum of these fungi (Rubini et al., 2005; de Souza et al., 2006; Bailey et al., 2008; Mejia et al., 2008).

In coffee systems, research conducted during this period advanced the application of *Trichoderma* as a biofertilizer, demonstrating its ability to enhance nutrient availability through the degradation of lignocellulosic residues primarily cellulose and hemicellulose present in crop by-products (Otieno et al., 2003; Eida et al., 2011). Studies also confirmed its natural occurrence in coffee agroecosystems (Gama et al., 2006; Sette et al., 2006; Fujii and Takeshi, 2007; Mulaw et al., 2010; Eida et al., 2011), supporting its ecological compatibility. Parallel findings showed its antimicrobial potential against coffee pathogens (Otieno et al., 2003; Sette et al., 2006), consolidating the dual role of *Trichoderma* as both a biofertilizer and biocontrol agent. Collectively, these advances fueled the surge of citations between 2006 and 2009, marking a period of consolidation and international recognition.

After 2010, the literature increasingly emphasized ecological functions and biotechnological applications. Studies explored its role in the biodegradation of coffee residues and lignocellulosic biomass conversion into fermentable sugars via cellulases, hemicellulases, and mannanases (Agrawal et al., 2011; Dessie et al., 2018; Juliastuti et al., 2018; Iswanto et al., 2019). These contributions not only improved the chemical characterization of coffee waste as a growth substrate but also expanded the biotechnological versatility of *Trichoderma* spp. (Kumar et al., 2018). In addition, its ecological plasticity was evidenced by tolerance to caffeine, which, rather than inhibiting growth, enhanced its mycoparasitic activity (Sugiyama et al., 2016). Such adaptations underpin its ability to occupy diverse ecological niches, ranging from the rhizosphere to endophytic tissues (Arias and Abarca, 2014; Bongiorno et al., 2016).

More recent research has transitioned toward molecular characterization, identifying gene families underlying its multifaceted functions (Jingade et al., 2018; Mulatu et al., 2022; Choez-Guaranda et al., 2023). Despite promising results, a disconnect between molecular advances and field-level applications persists. The sustained decline in citations after 2008, despite increasing publication numbers, reflects a fragmentation of research lines and highlights the need for integrative frameworks that link molecular insights with applied outcomes in tropical cropping systems.

#### 3.3.2 Scientific contributions by geographic regions

In terms of regional scientific production, Indonesia leads in publications on agricultural applications of *Trichoderma*. This emphasis reflects the strategic importance of coffee and cacao to the national economy and the urgent need for sustainable crop management strategies. Research in this region has primarily focused on three thematic axes: (i) development of integrated biological control systems for key pests and diseases, (ii) optimization of plant growth through bio-stimulation, and (iii) sustainable transformation of agricultural residues.

Innovative applications have broadened the scope of *Trichoderma* research. For instance, Amin et al. (2014) reported, for the first time, its use as an entomopathogen, achieving mortality rates of 84–96% in eggs of *Conopomorpha cramerella*. Similarly, Anggraeni et al. (2014) demonstrated the dual role of *Dolichoderus thoracicus* ants in pest control and in dispersing *Trichoderma*. to suppress *Phytophthora palmivora* in cacao. The biocontrol efficacy of *Trichoderma* spp. has been widely documented against major phytopathogens of the region. In cacao, it has proven effective against *Ceratobasidium theobromae*, *Phytophthora* spp., and *Lasiodiplodia theobromae* (Rosmana et al., 2015, 2019; Sriwati et al., 2015; Kuswinanti et al., 2019; Khairillah et al., 2021), as well as against *Candida albicans* and *Hemileia vastatrix* in coffee (Khairillah et al., 2021; Wulansari et al., 2023). Recent field trials confirm these promising outcomes: Umrah et al. (2024) reported that liquid formulations with spore densities of 9.76 × 10^8^ reduced the incidence of *P. palmivora* and *O. theobromae* by up to 77%, while Harni et al. (2023) observed disease reductions of 8.6–19.5%, coupled with yield increases of 12.2–37.0%.

Synergistic strategies have also been explored to enhance biocontrol efficacy. Rosmana et al. (2023) showed that co-inoculation with other endophytes boosted *T. asperellum* colonization in seedlings by 4–68 times, significantly suppressing leaf anthracnose caused by *Colletotrichum gloeosporioides.* Similarly, Sofiana et al. (2025) identified *T. viride* and *T. asperellum* strains that achieved 79% *in vitro* inhibition and 77% *in vivo* control of cacao pod rot caused by *Phytophthora palmivora*. Beyond biocontrol, studies have demonstrated additional applications in the rehabilitation of aging plantations and the production of high-quality compost from crop residues (Thaha et al., 2020; Palad et al., 2025). In coffee, applications at 3% concentrations significantly increased plant height (+17.6%), leaf number (+31.9%), and stem diameter (+49.1%) (Soleh et al., 2025). These findings align with improvements in leaf area, stem development, and graft success reported by Ala et al. (2020). Furthermore, Kartikal et al. (2023) confirmed that combining *Trichoderma* with arbuscular mycorrhizal fungi significantly enhanced nitrogen and phosphorus uptake in peat soils.

In Brazil, research development was driven by the need to combat *Moniliophthora perniciosa*, the causal agent of witches’ broom, one of the most devastating cacao diseases (De Marco et al., 2000; Rubini et al., 2005; Medeiros et al., 2010). Field and laboratory studies elucidated the morphological structures of the genus and its mechanisms of action - mycoparasitism and antibiosis - underpinning its role in biocontrol (Inglis et al., 1999; de Melo and Faull, 2004; De Souza et al., 2008). Research later expanded to other cacao diseases, demonstrating its efficacy against black pod caused by *Phytophthora palmivora* and vascular wilt caused by *Ceratocystis cacaofunesta* (Hanada et al., 2008, 2009, 2010; de Sousa et al., 2021). In coffee, investigations focused on endophyte–host interactions and biocontrol potential against *Hemileia vastatrix* and *Meloidogyne incognita,* showing that *Trichoderma* provides protection against pests and diseases while enhancing plant growth (Bongiorno et al., 2016; Del Carmen et al., 2021; De Sousa et al., 2022; Alves et al., 2023).

In contrast to Indonesia and Brazil, where research emerged in response to local agricultural challenges, the United States ranks third despite not being a major producer of coffee or cacao. This prominence can be attributed to strong technical capacity, advanced laboratory infrastructure, and international collaborations enabling both basic and applied research on pathogens and biocontrol strategies, often using samples sourced from tropical regions. U. S.-based studies have made critical contributions to understanding *Trichoderma–plant* interactions, including endophytic colonization of cacao and coffee tissues—such as glandular trichomes (Bailey et al., 2008, 2009) and the production of antifungal metabolites, notably nonanoic acid, which inhibits spore germination and mycelial growth of *M. roreri* and *C. perniciosa* (Aneja et al., 2005). Additionally, work on formulations has improved conidial germination and biocontrol efficacy under field conditions (Crozier et al., 2015). New species and strains with biotechnological potential have also been characterized (Samuels et al., 2006), alongside transcriptomic studies revealing host–fungus gene expression during colonization (Bailey et al., 2006), thereby deepening the molecular understanding of biocontrol efficiency.

In cacao-producing countries such as Peru and Ecuador, moniliasis (*M. roreri*) represents the primary threat to crop biodiversity, driving research into the mechanisms of *Trichoderma* action against this pathogen (Díaz-Valderrama et al., 2020; El Salous et al., 2020; Leiva et al., 2020; Chavez-Jalk et al., 2022; Chochocca et al., 2022). Emerging studies have also documented its role in soil bioremediation, particularly in cadmium-contaminated systems (Cayotopa-Torres et al., 2021; Malca-Cerna et al., 2025), signaling an important frontier for integrating agricultural sustainability with environmental remediation.

#### 3.3.3 Research patterns in coffee and cacao crops

Research on *Trichoderma* in coffee and cacao agroecosystems has converged on several thematic axes, including plant physiology, soil–microbe–plant interactions, biological control, biodiversity, and molecular characterization. Studies on microbial diversity in the rhizosphere have consistently identified *Trichoderma* as an endemic inhabitant of these agroecosystems (Mulaw et al., 2010, 2013; Arias and Abarca, 2014; Bustamante et al., 2021; De Sousa et al., 2022). These investigations emphasize the critical role of the microbiome in maintaining plant health, enabling host plants to withstand biotic challenges and abiotic stresses (De Sousa et al., 2022).

The soil–microorganism–plant interaction provides dual benefits: enhancing resistance against pathogens and environmental stressors, while also stimulating growth through root colonization and activation of systemic defenses (Assefa et al., 2021; Del Carmen et al., 2021). For example, Escudero-Leyva et al. (2023) demonstrated that endophytic *Trichoderma* isolates not only promoted growth in coffee plants but also antagonized *Mycena citricolor,* the causal agent of American leaf spot. Across studies, mycoparasitism and antibiosis remain the most commonly reported mechanisms of antagonism underlying biocontrol efficacy (Leiva et al., 2020).

In Peru, the first report of antagonistic potential against *Moniliophthora roreri* (cacao frosty pod rot) highlighted the activity of several species, including *T. parareesei, T. lentiforme, T. orientale, T. asperelloides, T. inhamatum, T. zelobreve, T. afarasin, T. ghanense, T. rifaii, and T. breve* (Leiva et al., 2022). Strains belonging to the *Harzianum* clade showed the highest levels of mycoparasitism, antibiosis, and overall antagonistic potential, surpassing isolates from the *Longibrachiatum* and *Hamatum* clades. Biocontrol assays documented up to 100% mycoparasitism and approximately 60% antibiosis, underscoring the promise of *Trichoderma* spp. as a sustainable tool for cacao producers (Leiva et al., 2020).

Bibliometric analysis of the most influential authors and cited works highlights priority thematic areas. Samuels has led taxonomic and applied studies on *Trichoderma stromaticum* and related species, with particular emphasis on their role in controlling witches’ broom (*Moniliophthora perniciosa*) (Samuels et al., 2000, 2012; Samuels and Ismaiel, 2009). Pomella validated the field application of *Trichoderma stromaticum* in Brazilian production systems, integrating it into compatible management and fertilization practices (De Souza et al., 2008). Krauss explored the efficacy of antagonist mixtures in integrated management of frosty pod rot, witches’ broom, and black pod (*Phytophthora* spp.), demonstrating the influence of environmental and formulation factors on biocontrol success (Krauss and Soberanis, 2001, 2002). Although with fewer publications, Bae made a highly impactful contribution by demonstrating that endophytic strains of *Trichoderma* promote growth and enhance drought tolerance in *Theobroma cacao*, supported by molecular evidence of host–endophyte interactions (Bae et al., 2009).

Collectively, the most cited works reflect three predominant research trajectories: (i) functional endophytism and stress tolerance in cacao, as exemplified by *T. hamatum* DIS 219b, which delayed drought responses and altered host gene expression (Bailey et al., 2006; Bae et al., 2009); (ii) endophytic and community-level biocontrol against major pathogens, highlighting mechanisms of antagonism, antibiosis, and mycoparasitism (Rubini et al., 2005; Bailey et al., 2008; Mejia et al., 2008; Hanada et al., 2010); and (iii) taxonomic and phylogenetic foundations supporting the selection of effective biocontrol agents, including revisions of the *T. koningii* complex and *Hypocrea rufa/T. viride* lineages (Samuels et al., 2006). In coffee, recent studies reveal that prolonged monocultures alter soil chemical properties and microbial communities (Zhao et al., 2018), while endophytic isolates with antimicrobial potential have been reported (Sette et al., 2006), underscoring the transferability of endophytic biocontrol strategies across both crops.

#### 3.3.4 Advances in ecological and functional knowledge

Research on *Trichoderma* in coffee and cacao agroecosystems has evolved from basic taxonomic descriptions to a more integrative understanding of its ecological roles and functional mechanisms. This transition has been driven by the incorporation of molecular, physiological, and ecological approaches, enabling the characterization not only of species diversity but also of their ability to establish rhizospheric, epiphytic, and endophytic associations with host plants (Yadav et al., 2019; Lu et al., 2022; Waqar et al., 2024).

The ecological versatility of the genus is evident in its frequent occurrence in the rhizosphere and its ability to colonize intra- and intercellular spaces of plant tissues (Lorito et al., 2010; Sánchez Hernández et al., 2018; Choez-Guaranda et al., 2023). This colonization plasticity allows *Trichoderma* to significantly modulate host physiology, enhancing systemic activation of salicylic acid (SA) - and jasmonic acid (JA) - dependent defense pathways, while simultaneously improving nutrient acquisition through the stimulation of membrane transporters and mineral solubilization (Guzmán-Guzmán et al., 2025).

Functionally, these processes translate into relevant agronomic benefits, including enhanced plant growth (Martínez-De-Jesús et al., 2025), improved uptake of essential nutrients (Del Carmen et al., 2021; Perea-Rojas et al., 2025), and activation of resistance mechanisms against pathogens and abiotic stressors (Guzmán-Guzmán et al., 2025). In coffee, endophytic strains such as *Trichoderma harzianum* and *Trichoderma asperellum* have been reported to improve phosphorus and iron absorption while inducing resistance against *Hemileia vastatrix* and *Mycena citricolor* (Del Carmen et al., 2021; Escudero-Leyva et al., 2023). In cacao, *Trichoderma hamatum* (strain DIS 219b) has been shown to enhance seedling development and delay drought responses, maintaining photosynthetic activity and water balance for longer periods (Bae et al., 2009). Similarly, co-inoculation of *Trichoderma asperellum* with arbuscular mycorrhizal fungi significantly improved growth and reduced Phytophthora megakarya incidence under field conditions (Tondje et al., 2007).

In terms of biocontrol, *Trichoderma* spp. species act through multiple mechanisms, including mycoparasitism, antibiosis, and competition for nutrients and space (Tyśkiewicz et al., 2022; Waqar et al., 2024). This functional diversity has demonstrated efficacy against major pathogens of coffee and cacao. In Ethiopia, for example, *Trichoderma asperellum* AU131 and *Trichoderma longibrachiatum* AU158 inhibited *Fusarium xylarioides*, the causal agent of coffee wilt disease, with suppression rates exceeding 80% and field biocontrol effectiveness of approximately 84% (Mulatu et al., 2023). Similarly, in cacao, extracts of *Trichoderma spirale*, *Trichoderma harzianum,* and *Trichoderma reesei* have shown antifungal activity against *Moniliophthora perniciosa* and *Moniliophthora roreri* (Choez-Guaranda et al., 2023). Native Peruvian isolates, particularly those belonging to the Harzianum clade, have also exhibited strong mycoparasitism and antibiosis against *Moniliophthora roreri*, with field control rates above 70% (Leiva et al., 2020, 2022). More recently, certain strains have been shown to inhibit both *Moniliophthora roreri* and *Moniliophthora perniciosa* simultaneously through the production of chitinolytic enzymes and volatile organic compounds, achieving inhibition rates exceeding 90% (Garcés-Moncayo et al., 2025).

#### 3.3.5 Biotechnological applications

The transition toward sustainable agriculture has accelerated the development of biological products as viable alternatives to conventional agrochemical inputs. Within this context, species of the genus *Trichoderma* spp. represent one of the most validated and widely adopted microbial technologies for crop protection and soil health management. Their effectiveness in biofungicide formulations has been consistently demonstrated across diverse systems. For instance, two commercial prototypes - wettable powder and water-dispersible granules - based on *Trichoderma asperellum* AU131 and *Trichoderma longibrachiatum* AU158 inhibited more than 80% of *Fusarium xylarioides* and achieved biocontrol efficiencies above 77% under field conditions (Mulatu et al., 2023).

Complementary evidence highlights the multifunctionality of *Trichoderma* spp. *Trichoderma asperellum* significantly reduced populations of *Meloidogyne* nematodes in established coffee plantations, thereby improving soil health and enhancing associated microbial diversity (Saikai et al., 2023). Studies from Cameroon and Ethiopia further reported endophytic *Trichoderma* strains colonizing coffee tissues and parasitizing *Hemileia vastatrix*, the causal agent of coffee leaf rust, suggesting their potential as protective agents against this economically devastating disease (Del Carmen et al., 2021).

In cacao, field trials demonstrated that suspensions of *Trichoderma harzianum* and *Trichoderma virens* reduced the incidence of pod rot caused by *Phytophthora palmivora* by up to 48%, validating their practical applicability in production systems (Sriwati et al., 2019). However, the success of these biofungicides depends critically on formulation stability, shelf-life, and the optimization of delivery systems (Kumar et al., 2018, 2023).

Beyond biocontrol, the biotechnological potential of *Trichoderma* spp. is evident in its role as a cellulolytic fungus capable of accelerating the bioconversion of agricultural residues into high-quality biofertilizers. In coffee systems, berry husks - which account for up to 80% of processed fruit biomass—have been valorized through composting with *Trichoderma harzianum.* This process enhances organic matter decomposition, improves nutrient release, mitigates environmental contamination, and enriches soil fertility (Nduka et al., 2017; Muzakir et al., 2024). The incorporation of *Trichoderma* spp. into coffee composts has also been shown to increase seedling vigor traits such as leaf area and grafting success, confirming its dual role as decomposer and biostimulant (Ala et al., 2020).

Similarly, in cacao systems, the inoculation of composted pod husks and pruning residues with *Trichoderma* spp. increased organic carbon content, macronutrient availability (N, P, K), and pH, producing biocomposts with improved agronomic value (Thaha et al., 2020). Experimental trials further demonstrated that cacao seedlings grown with *Trichoderma*-enriched compost exhibited significant gains in height, stem diameter, and chlorophyll content (Osmar, 2020).

#### 3.3.6 Knowledge gaps and future perspectives

Despite substantial advances in understanding the ecology and functional roles of *Trichoderma* in coffee and cacao agroecosystems, most studies remain concentrated on well-established mechanisms such as mycoparasitism, antibiosis, and plant growth promotion. Critical gaps persist in emerging and strategic areas that could significantly expand its applications in sustainable agriculture.

One promising yet underexplored avenue is the entomopathogenic potential of *Trichoderma.* Preliminary evidence suggests its capacity to affect insect pests of economic importance. For instance, *Trichoderma* spp. reduced survival of cacao pod borer (*Conopomorpha cramerella*) eggs by 84–89% (Amin et al., 2014). Similarly, antagonistic activity has been suggested against *Pseudococcidae* (root mealybugs) in coffee (Gil et al., 2023), though results remain inconclusive. While still at an early stage, these findings open new perspectives for positioning *Trichoderma* as a multifunctional biocontrol agent within integrated pest management programs.

Another emerging field concerns the role of *Trichoderma* in heavy-metal bioremediation. In cacao, cadmium (Cd) accumulation in beans represents a critical limitation for chocolate production and international trade, creating demand for microbial strategies that mitigate its uptake. *In vitro* assays have shown that species such as *Trichoderma brevicompactum, Trichoderma harzianum,* and *Trichoderma spirale* can reduce cadmium concentrations by 83, 67, and 66%, respectively (Cayotopa-Torres et al., 2021). However, *in vivo* studies present a more complex scenario: while some strains reduce Cd bioaccumulation, others – such as *Trichoderma orientale* BLPF1-C1—have been reported to exacerbate cadmium uptake to phytotoxic levels (Malca-Cerna et al., 2025). These discrepancies underscore the host–soil–microbe context dependency of *Trichoderma* efficacy in phytoremediation. Future research must elucidate the physiological and molecular mechanisms underlying these interactions and prioritize the selection of strains with consistent and safe remediation profiles.

A third area relates to crop residue bioconversion, one of the earliest research lines involving *Trichoderma* in coffee and cacao systems. Early studies demonstrated its capacity to accelerate organic matter decomposition, enhance soil microbial populations, and improve degraded soils, while some species showed specificity for degrading compounds such as caffeine (Tauk, 1985; Roussos et al., 1995; Thaha et al., 2020). However, despite its initial prominence, research in this field has declined markedly in recent decades. This decline contrasts with the urgent need for sustainable strategies to manage agricultural residues, highlighting the importance of reinvigorating this research line through multidisciplinary approaches that integrate microbial biotechnology with circular economy principles.

Overall, bridging these gaps will require moving beyond classical biocontrol paradigms and embracing integrative frameworks that link *Trichoderma’s* ecological versatility with biotechnological innovations. Doing so could position this genus not only as a cornerstone of plant protection but also as a multifunctional ally for sustainability, resilience, and environmental stewardship in tropical agroecosystems.

#### 3.3.7 Limitations of the study

This study integrated a bibliometric analysis with a systematic review to characterize the ecological and functional roles of the genus *Trichoderma* in coffee and cacao agroecosystems. Nonetheless, several limitations must be acknowledged when interpreting the results. First, the bibliometric analysis was restricted to two major databases (Scopus and Web of Science). While this ensured the quality and relevance of the included publications, it may have excluded scientific contributions not indexed in these repositories or published in regional journals. In addition, data curation relied on thematic filters that could have overlooked interdisciplinary studies with indirect yet relevant applications to coffee and cacao systems.

Regarding the systematic review, methodological heterogeneity among the selected studies – including differences in field conditions, experimental scales, *Trichoderma* species, and host crop varieties – limits the possibility of quantitative comparisons or meta-analyses. Furthermore, much of the available evidence derives from laboratory or greenhouse trials, which restricts the extrapolation of findings to field conditions and highlights the need for cautious interpretation.

Another constraint is that most studies focus on classical mechanisms such as mycoparasitism, antibiosis, growth promotion, and induced resistance, while emerging areas – including heavy-metal bioremediation, crop residue bioconversion, and entomopathogenic potential – remain underrepresented and largely preliminary. Finally, the variability in the performance of native strains under different edaphoclimatic conditions underscores the importance of validating these findings across diverse and representative environments.

Taken together, these limitations emphasize the need for future research that combines interdisciplinary approaches with large-scale field trials. Such efforts will be essential to assess the consistency, safety, and practical applicability of *Trichoderma* as a strategic microbial resource for the sustainability and resilience of tropical agroecosystems.

## 4 Conclusion

The integrative analysis of the literature highlights the ecological and functional significance of the genus *Trichoderma* in coffee and cacao agroecosystems. This microorganism exhibits remarkable versatility in colonizing multiple niches – including the rhizosphere, endosphere, and phyllosphere—where it interacts directly with host plants while modulating the dynamics of associated microbial communities. Such multifunctionality positions *Trichoderma* as a stabilizing organism that enhances nutrient availability, accelerates decomposition processes, improves tolerance to abiotic stress, and balances microbial interactions, thereby contributing to the ecological resilience of tropical cropping systems.

The evidence synthesized confirms the importance of well-established mechanisms—mycoparasitism, antibiosis, systemic resistance induction, and plant growth promotion—which have been widely validated for pathogen suppression and productivity enhancement. In addition, emerging functions broaden its biotechnological spectrum, including heavy-metal bioremediation (particularly cadmium in cacao), crop residue bioconversion, and its nascent potential as an entomopathogen against economically relevant pests. These findings reinforce the multifunctionality of *Trichoderma*, and its strategic value for sustainable agriculture.

Nevertheless, limitations and contradictions persist. While some strains reduce cadmium accumulation or exhibit strong efficacy against pests, others may trigger adverse outcomes, such as excessive metal bioaccumulation or inconsistent responses depending on crop genotype and soil conditions. Furthermore, the declining research attention to areas such as agricultural residue management reveals a critical gap in a field highly relevant to circular economy practices.

Harnessing the full potential of *Trichoderma* will require expanded field-based research and molecular approaches to achieve a more precise understanding of its interaction mechanisms. Equally important are the identification and selection of strains with consistent performance, the development of stable and scalable formulations, and the integration of this microbial resource into holistic strategies for integrated pest management, agroecological sustainability, and circular economy innovation.
