# Supplementary figures and images for: Ecology and functions of Trichoderma in coffee and cocoa agroecosystems: bibliometric and systematic insights for sustainable agriculture
Source: Front Microbiol. 2025 Nov 28;16:1717484. doi: 10.3389/fmicb.2025.1717484 (PMC12698567; doi:10.3389/fmicb.2025.1717484)

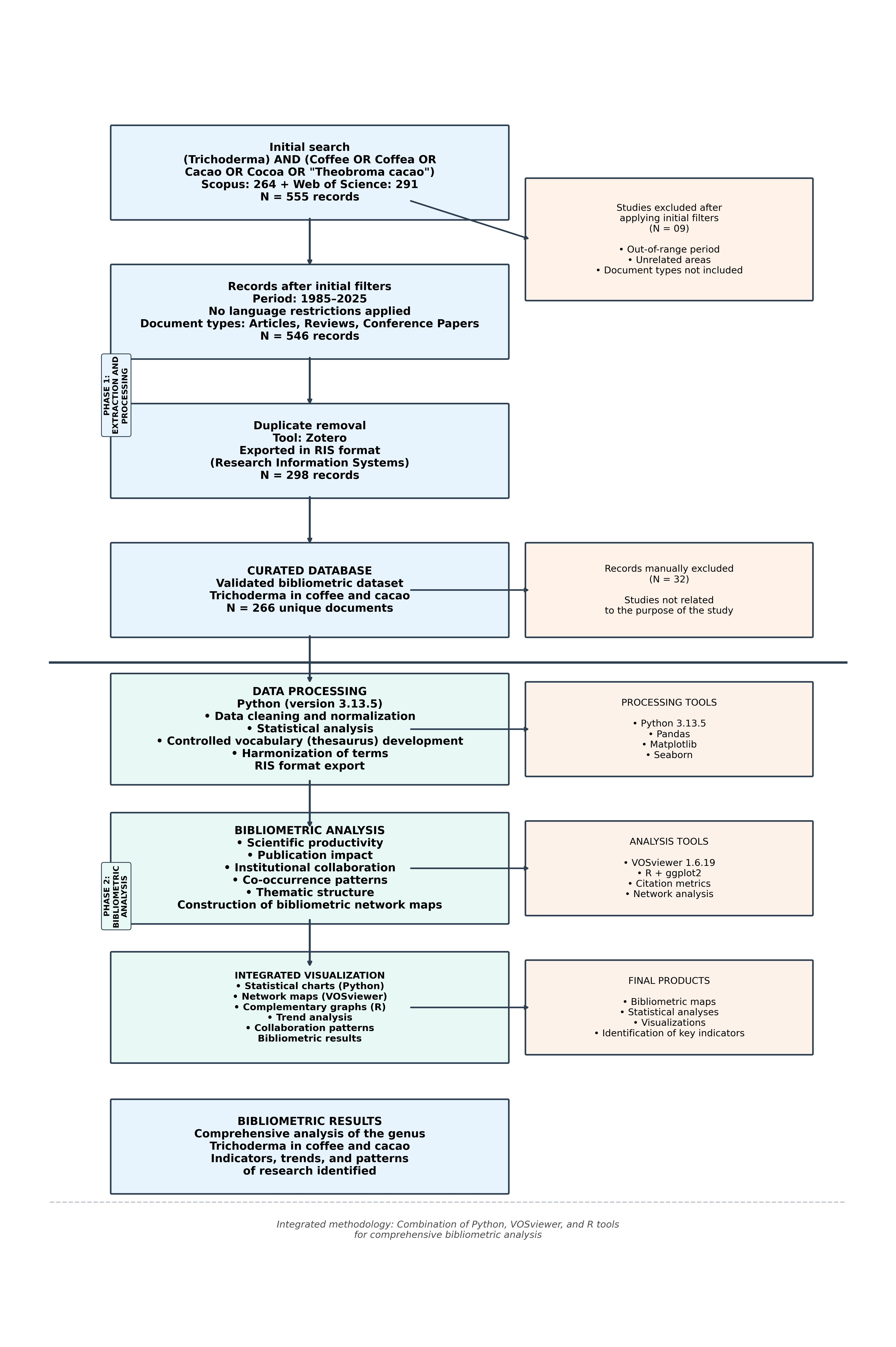

Supplement: Supplementary file 1 [file Image_1.JPEG]
